# Surface Characterization of MoS_2_ Atomic Layers Mechanically Exfoliated on a Si Substrate

**DOI:** 10.3390/ma13163595

**Published:** 2020-08-14

**Authors:** Mirosław Krawczyk, Marcin Pisarek, Robert Szoszkiewicz, Aleksander Jablonski

**Affiliations:** 1 Laboratory of Surface Analysis, Institute of Physical Chemistry, Polish Academy of Sciences, Kasprzaka 44/52, 01-224 Warsaw, Poland; mpisarek@ichf.edu.pl (M.P.); ajablonski@ichf.edu.pl (A.J.); 2 Biological and Chemical Research Centre, Faculty of Chemistry, University of Warsaw, Żwirki Wigury 101, 02-089 Warsaw, Poland; rszoszkiewicz@chem.uw.edu.pl

**Keywords:** molybdenum disulfide, Auger electron spectroscopy, scanning electron microscopy, surface composition and morphology, elastic-peak electron spectroscopy, electron inelastic mean free path

## Abstract

Mo disulfide overlayers with the thickness exceeding 1.77 nm were obtained on Si substrates through mechanical exfoliation. The resulting Mo disulfide flakes were then analyzed ex situ using combination of Auger electron spectroscopy (AES), elastic-peak electron spectroscopy (EPES) and scanning electron microscopy (SEM) in order to characterize their surface chemical composition, electron transport phenomena and surface morphology. Prior to EPES measurements, the Mo disulfide surface was sputter-cleaned and amorphized by 3 kV argon ions, and the resulting S/Mo atomic ratio varied in the range 1.80–1.88, as found from AES measurements. The SEM images revealed single crystalline small-area (up to 15 μm in lateral size) Mo disulfide flakes having polygonal or near-triangular shapes. Such irregular-edged flakes exhibited high crystal quality and thickness uniformity. The inelastic mean free path (IMFP), characterizing electron transport, was evaluated from the relative EPES using Au reference material for electron energies *E* = 0.5–2 keV. Experimental IMFPs, *λ*, determined for the AES-measured surface compositions were approximated by the simple function *λ* = *kE^p^*, where *k* = 0.0289 and *p* = 0.946 were fitted parameters. Additionally, these IMFPs were compared with IMFPs resulting from the two methods: (i) present calculations based on the formalism of the Oswald et al. model; (ii) the predictive equation of Tanuma et al. (TPP-2M) for the measured Mo_0.293_S_0.551_C_0.156_ surface composition (S/Mo = 1.88), and also for stoichiometric MoS_2_ composition. The fitted function was found to be reasonably consistent with the measured, calculated and predicted IMFPs. We concluded that the measured IMFP value at 0.5 keV was only slightly affected by residual carbon contamination at the Mo disulfide surface.

## 1. Introduction

Recently, transition-metal dichalcogenides (TMDCs) have attracted substantial research interest due to their electronic, optical and tribological properties. Molybdenum disulfide (MoS_2_) is one of the TMDCs that has been successfully exfoliated into a very stable single monolayer or few layers-thick material [1]. In particular, atomically thin MoS_2_ with the structural configuration of two-dimensional and/or three-dimensional atomic layers has emerged as the most promising low-dimensional nanomaterial for novel applications in nanoelectronics [2], photovoltaics [3], valleytronics [4], and hydrogen evolution reaction catalysis [5,6,7].

For the fabrication of MoS_2_-based nano-materials, mechanical exfoliation [1] of bulk MoS_2_ crystals is a typical method to isolate and transfer high-quality single- and few-layer MoS_2_ onto relevant substrates. However, this method is difficult for large-scale production due the lack of control in the thickness, shape, size, and position of molybdenum disulfide layers. Commercial applications require thin film deposition techniques for large-area (cm-scale) growth of continuous films on wafer substrates. Such deposition processes that allow direct synthesis on substrates should be highly scalable and should be an integral part of microelectronic manufacturing. Gas-phase methods like chemical vapor deposition (CVD) [8] and atomic layer deposition (ALD) [9] hold the greatest promise in meeting these requirements.

It is well known that surface properties (composition, morphology, electron transport, etc.) of thin molybdenum disulfide layers play a decisive role for the aforementioned technological applications. The accurate quantification of Mo disulfide surface analysis using electron spectroscopies, e.g., Auger electron spectroscopy (AES), requires knowledge of the electron inelastic mean free path (IMFP) value, which is still unavailable. IMFP, however, characterizes electron transport properties in the surface region of each solid. It has been postulated [10] that the IMFPs that are in agreement with the definition can be calculated from experimental optical data [10,11,12,13,14] or they can be derived from elastic-peak electron spectroscopy (EPES) experiments [15,16]. It has also been demonstrated that EPES is a convenient tool for the determination of the IMFP values from studies of overlayers with thickness in the range of nanometers [17]. Such experiments were recently performed to evaluate IMFPs in the yttrium oxide overlayers grown on yttrium substrate [18]. The IMFPs in materials, for which no IMFP calculations or EPES experiments have been conducted, can be also estimated with unknown accuracy from the TPP-2M predictive equation [19]. This universal equation was developed by Tanuma, Powell and Penn from an analysis of their computed IMFPs for 27 elements and 14 organic compounds. It is based on the Bethe equation for inelastic electron scattering in matter but with some modifications to describe the IMFP dependence on energy for energies less than 200 eV. The TPP-2M IMFPs, similarly to those calculated from optical data, refer to the bulk of a solid.

In the case of the EPES method, it is known that the elastic electron backscattering probability is related to the intensity of the so-called elastic peak in the energy spectrum. Contrarily, the elastic backscattering probability can also be derived from theoretical models describing the electron transport in solids. These models can provide the relation between the elastic peak signal intensity and the IMFP which is the basis for determination of this parameter. Dubus et al. [20] critically compared and discussed the assumptions that simplify different theoretical models of electron transport. As a result of this review, the Oswald–Kasper–Gaukler (OKG) model [21] was selected as being suitable for analytical applications. The formalism of the OKG model was further modified by including relativistic effects [20] and was tested in the analytical practice of EPES [22]. It has been found [23] that the accuracy of the IMFPs calculated from the modified model is comparable with IMFPs from the Monte Carlo simulations of electron trajectories in elemental solids. This model extended to alloys and compounds [24] is applied in the present work for determining the IMFP in Mo disulfide from the elastic peak intensity.

The aim of this work was to examine the surface properties (surface composition, surface morphology, and electron transport) of molybdenum disulfide layer obtained on Si substrate in controlled conditions. For the first time, according to our knowledge, we evaluate the IMFPs in thin Mo disulfide overlayer with different surface stoichiometry for electron transport in the range of 0.5–2 keV, using the relative EPES measurements. These measurements are preceded by AES and scanning electron microscopy (SEM) examination of the surface composition and morphology of the molybdenum disulfide overlayer.

## 2. Experimental

*Samples:* MoS_2_ was mechanically exfoliated by the Scotch-tape method [25] from commercially available bulk MoS_2_ crystals (SPI Supplies, West Chester, PA, USA, cat. no. #429MM-AB), and then transferred on fine polished and basically undoped <111> Si crystals with resistivity of more than 10,000 Ω·cm (ITME, Warsaw, Poland). This preparation procedure is essentially identical to that used earlier to obtain thick MoS_2_ flakes [26,27].

For determination of the electron IMFP in the molybdenum disulfide sample, gold foil (0.1 mm–thick, 99.9975%+ purity, bought from Alfa Aesar GmbH and Co KG, Karlsruhe, Germany) was used as a reference material.

*Methods of surface characterization and pretreatments:* Surface characterization of molybdenum disulfide overlayer of Si substrate were performed using the two electron spectroscopies AES and EPES, and also SEM.

The AES characterization was performed using Microlab 350 spectrometer (Thermo Scientific, Waltham, MA, USA) with a spherical sector analyzer. The Auger spectra were recorded in the differential mode dN(E)/dE at the normal incidence of the primary electron beam of 5 keV. The spectra were quantified using the software Avantage (ver. 4.88, Thermo Fisher Scientific Inc., Waltham, MA, USA) for the Mo M_4.5_N_2.3_V (190 eV), S LMM (153 eV) and C KLL (275 eV) peak intensities [28]. Their Auger sensitivity factors extracted from this software package were 1.278, 4.758 and 0.614, respectively. The Auger spectra were obtained from the sample surfaces which were initially sputter-cleaned by 3 kV Ar^+^ ions (a maximal ion current was about 1.3 μA) rastered over a 3 × 3 mm^2^ surface area at an incidence angle of 30° with respect to surface normal. This sputter-cleaning was maintained for 30 s prior to EPES studies.

Elastic-peak intensities for both the Mo disulfide surface and the surface of Au reference material were also recorded using a Microlab 350 spectrometer at electron energies of 500, 1000, 1500 and 2000 eV. The Au reference material has been recently indicated as the preferable standard material [29] having the known energy dependence of the IMFP recommended for the EPES method [10]. In relative EPES experiments, the electron gun was located at the normal to the surface and the analyzer axis was located at 60° to the surface normal. The acceptance half-angle of the analyzer was 6°. The EPES procedure of relative measurements applied in the present work was already described in [16]. The electron energy dependence of the IMFP, characterizing electron transport, for the surface composition of the analyzed Mo disulfide sample was determined using the software package EPES [30] which allows elastic-peak spectra processing and Monte Carlo simulations of electron trajectories in solids.

Before the EPES measurements, the surfaces of Mo disulfide and Au samples were also cleaned by sputtering with 3 kV argon ions to remove surface contamination. After 30 s of sputtering, oxygen and carbon contaminants were entirely removed from the surface region of gold; however, some residual carbon contamination at the molybdenum disulfide surface was still detected by AES analysis.

Surface morphology of the Mo disulfide flakes was also studied by the Microlab 350 (Thermo Scientific, Waltham, MA, USA) apparatus, which is additionally equipped with SEM technique. The field emission gun (FEG) was used to recorded SEM images at beam energy 3 kV. At low excitation energy, real topography of the flake surfaces and their uniformity in terms of thickness could be observed. Moreover, the homogeneity of the chemical composition of the analyzed flakes was confirmed by performing chemical composition maps for the analyzed elements: S, Mo and Si using the same excitation energy.

## 3. Results and Discussion

### 3.1. Auger Electron Spectroscopy (AES) Characterization

**Figure 1:**
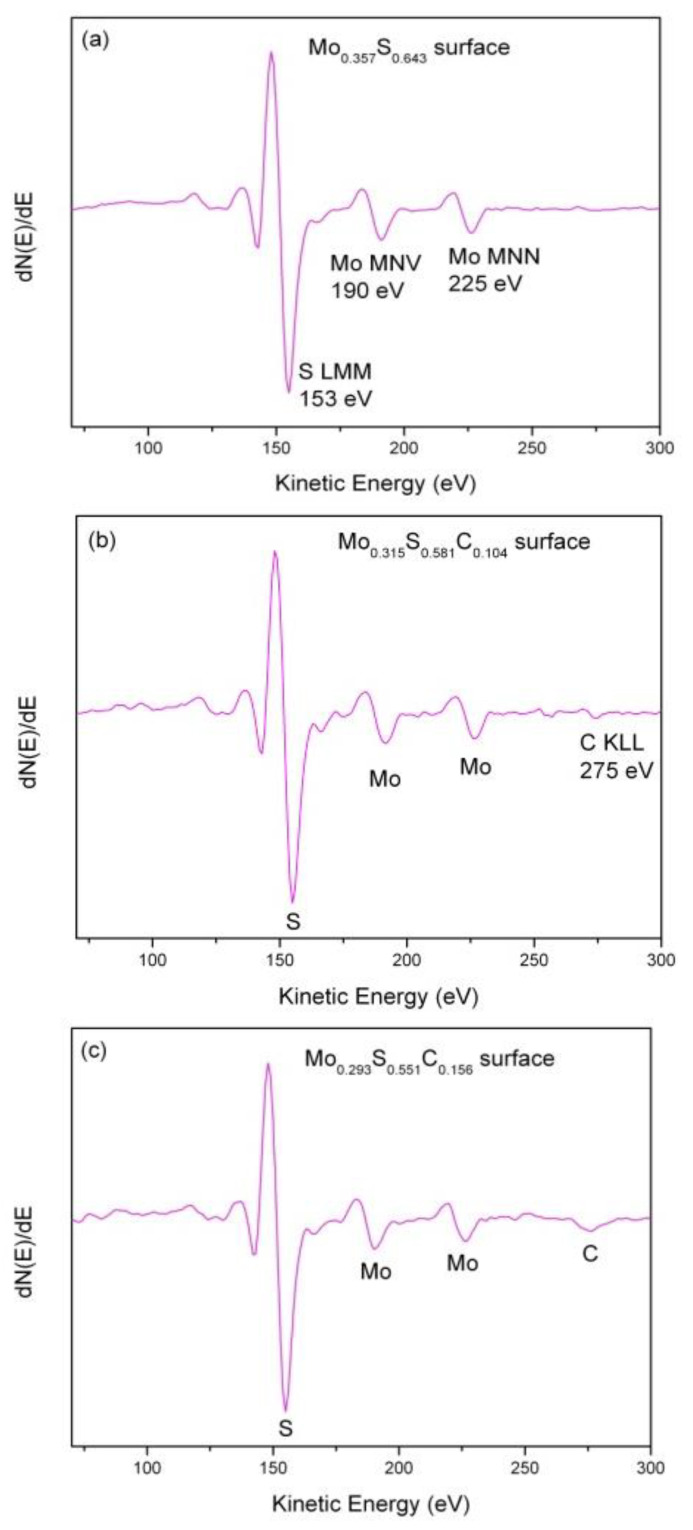
Auger electron spectroscopy (AES) spectra of the sputter-cleaned (3 kV Ar^+^ ions, 30 s) molybdenum disulfide flakes with increasing S/Mo ratio prior to elastic-peak electron spectroscopy (EPES) measurements: (**a**) S/Mo = 1.80; (**b**) S/Mo = 1.84; (**c**) S/Mo = 1.88.

The surface composition of molybdenum disulfide/silicon heterostructures was examined by AES analysis. Figure 1 shows the Auger spectra from the three Mo disulfide surfaces after 3 kV Ar^+^ ion sputtering for 30 s. This is a standard cleaning procedure to minimalize and/or remove surface contamination (i.e., removing 4.8 nm in depth of material) prior to EPES measurements. We note only molybdenum (the two prominent Auger MNV and MNN peaks at 190 and 225 eV, respectively) and sulfur (the Auger LMM peak at 153 eV) in Figure 1a while Figure 1b,c shows additionally carbon (the Auger KLL peak at 275 eV) at the sputter–cleaned surfaces. As a result of quantitative Auger analysis, a final elemental composition of each Mo disulfide surface can be expressed as Mo_0.357_S_0.643_, Mo_0.315_S_0.581_C_0.104_, and Mo_0.293_S_0.551_C_0.156_. The resulting S/Mo atomic concentration (AC) ratios are found to be 1.80, 1.84, and 1.88, respectively, and these values are smaller than the MoS_2_ value. There is an excellent agreement between our AES results and the recently reported result of quantitative X-ray photoelectron spectroscopy (XPS) analysis for the exfoliated (0001) MoS_2_ surface after annealing under ultrahigh vacuum (UHV) conditions [31]. In this case, a sulfur-deficient surface composition of MoS_1.8_ was found.

As shown in Figure 1b,c, the as-sputtered MoS_2_ surfaces still contain about 10–15 at.% of carbon contamination, most likely caused by mechanical exfoliation. In addition, there is the lack of any Si peaks originating from the substrate material in the AES spectra (Figure 1) recorded from Mo disulfide/Si heterostructures.

To estimate the thickness of the molybdenum disulfide (Mo_0.357_S_0.643_) or the molybdenum disulfide-carbon (Mo_0.315_S_0.581_C_0.104_, Mo_0.293_S_0.551_C_0.156_) overlayers determined by an AES analysis depth, we should know the IMFPs for Auger electrons emitted by S, Mo and C atoms at the kinetic energies 153 eV, 190 eV and 275 eV [28], respectively. The TPP-2M predictive formula implemented in the NIST Electron Inelastic-Mean-Free-Path Database (SRD 71) [32] were used to calculate these IMFPs in the considered layers. Among the three elements, the smallest IMFP value of about 0.59 ± 0.08 nm was found for 153 eV LMM electrons of sulfur while the largest value of IMFP is equal to 0.82 ± 0.12 nm for C KLL electrons at 275 eV. Thus, the Mo disulfide layer was found to be at least 1.77 nm thick under the present AES measurement conditions (95% of the measured Auger electrons originate from the near-surface region up to a depth of 3 IMFPs). If this layer consisted of carbon atoms, its thickness can be larger than the value 3 × 0.82 nm = 2.46 nm.

Influence of surface carbon on the EPES-determined IMFP for molybdenum disulfide will be explained later.

### 3.2. Scanning Electron Microscopy (SEM) Characterization

**Figure 2:**
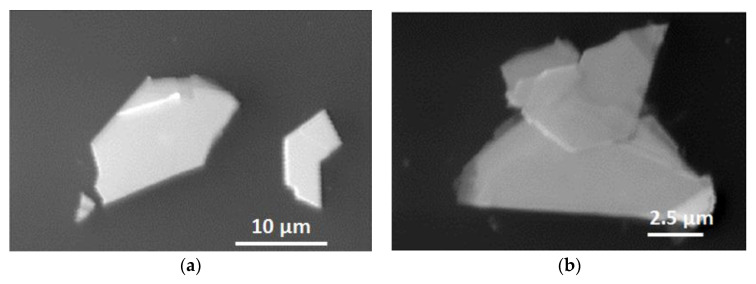

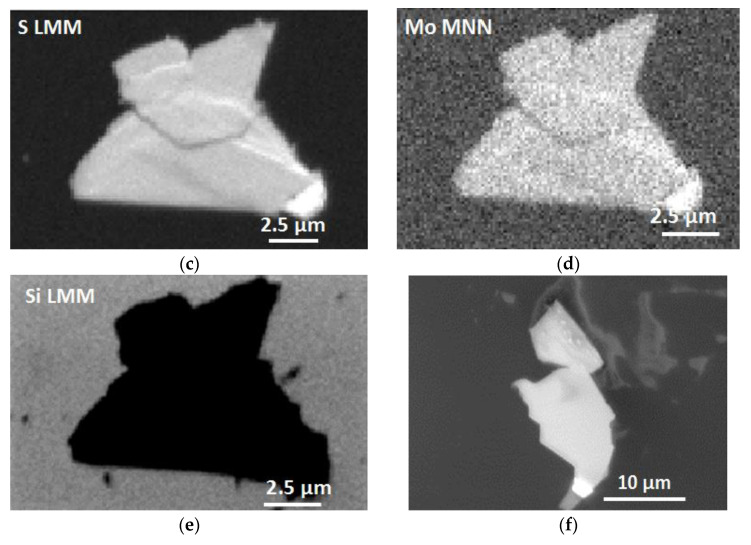
Scanning electron microscopy (SEM) images of the examined surfaces of Mo disulfide flakes exfoliated on silicon substrates with increasing S/Mo AC ratio: (**a**) S/Mo = 1.80; (**b**) S/Mo = 1.84; (**c**–**e**): Distribution images of elements for the Mo disulfide flake from (**b**): S (LMM) in (**c**), Mo (MNN) in (**d**) and Si (LMM) in (**e**) on the flake surface suggesting chemical homogeneity of considered surfaces; (**f**) another flake with S/Mo = 1.88.

The morphology of molybdenum disulfide flakes exfoliated on silicon substrates was characterized by SEM. We conducted this characterization on the same flakes, which were earlier analyzed by AES. Figure 2a shows the SEM images of Mo disulfide flakes exhibiting the bi-component Mo_0.357_S_0.643_ surface (the large flake seen in Figure 2a; AES/EPES-analyzed surface area is located around the center of this flake), and also both the three-component Mo_0.315_S_0.581_C_0.104_ (AES/EPES-analyzed surface area is located around the central zone of partially overlapping flakes seen in Figure 2b) and Mo_0.293_S_0.551_C_0.156_ (the large flake seen in Figure 2f; AES/EPES-analyzed surface area is located near the flake center) surfaces. The SEM analysis reveals single crystalline small-area (up to 15 µm in lateral size) of Mo disulfide flakes having polygonal or near-triangular shapes, as shown in Figure 2. It is important to note that such irregular-edged flakes exhibit high crystal quality and thickness uniformity. Moreover, the distribution images of the Auger S LMM, Mo MNN and Si LMM signals from the surface of the flake presented in Figure 2b shows unambiguously homogeneous distribution of S and Mo atoms (Figure 2c–e). It is noteworthy that in Figure 2e there is no signal from Si, which means that the analyzed surface area has the appropriate thickness for EPES analysis and homogeneous chemical composition. The S LMM and Mo MNN Auger signals implies the presence of molybdenum sulfide flake on the Si substrate.

### 3.3. Elastic-Peak Electron Spectroscopy (EPES) Characterization: Evaluation of the Electron Inelastic Mean Free Path (IMFP)

**Figure 3:**
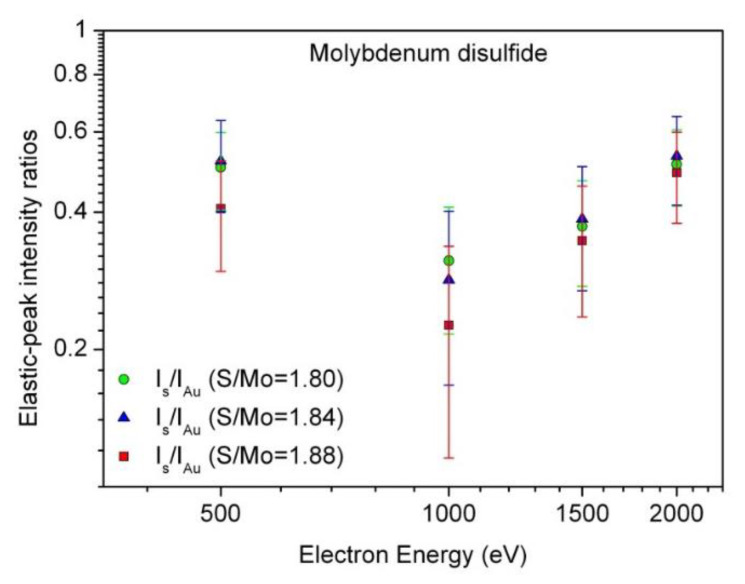
Energy dependence of the measured elastic-peak intensity ratios, *I_s_*/*I_Au_*, recorded from molybdenum disulfide surfaces with increasing S/Mo AC ratio: S/Mo = 1.80 (open circles); S/Mo = 1.84 (open triangles); S/Mo = 1.88 (open squares), and gold surfaces (α_in_ = 0°, α_out_ = 60° measured from the surface normal). Error bars for the *I_s_*/*I_Au_* ratios represent one standard deviation.

To evaluate the IMFPs and their energy dependence, the intensity of the measured elastic peaks were determined using the software EPES [30]. Figure 3 shows the ratios of measured elastic-peak intensity *I_S_* for the Mo disulfide flake surfaces with S/Mo atomic contents ratios ranging from 1.80 to 1.88 to the corresponding intensity *I_Au_* for the Au standard with error bars, as functions of electron energy.

We observe that the intensity ratios for the surface S/Mo ratio of 1.88 are the smallest in an entire energy range below 2000 eV. As follows from Figure 3, these values are smaller by 23–39% than those obtained for the S/Mo ratios of 1.8 and 1.84 in the energy range 500–1000 eV. The observed differences among the *I_s_*/*I_Au_* values can be explained mainly by the influence of surface inelastic-electron excitations. It is well known that the elastic-peak intensity for both the studied sample and the standard material is diminished by the surface excitations, and some deviations are possible if the surface excitations for both materials differ considerably. Generally, we may state that the surface excitation effects decrease with increasing electron energy. The surface excitation effects are not accounted for in the presently used software package EPES. Another source of the noticeable differences considered here can be due to the presence of carbon contamination in the surface region of Mo disulfide (with the S/Mo ratios of 1.84 and 1.88), especially at 500 eV. The measured elastic-peak intensities were finally used for determining the IMFP values in MoS_2_.

**Figure 4:**
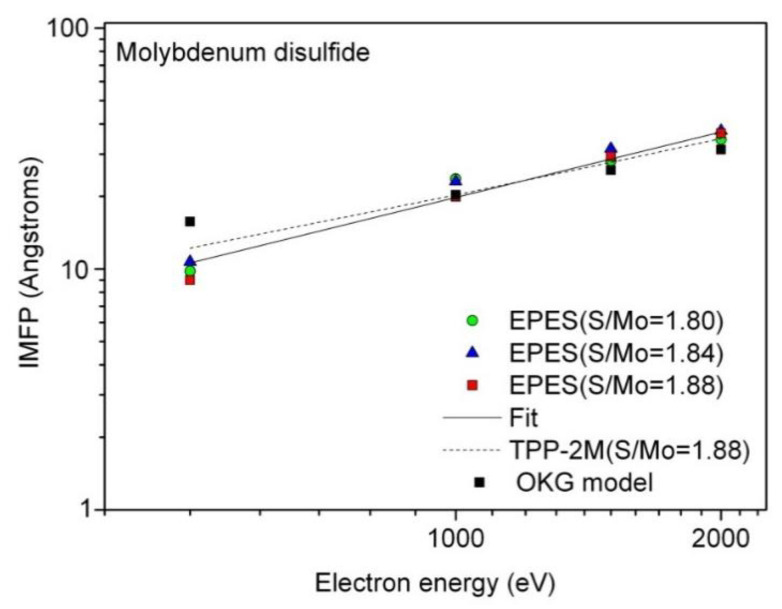
Energy dependence of the inelastic mean free path (IMFP) determined using the Au standard for the Mo_0.357_S_0.643_ (open circles, S/Mo = 1.8), Mo_0.315_S_0.581_C_0.104_ (open triangles, S/Mo = 1.84) and Mo_0.293_S_0.551_C_0.156_ (open squares, S/Mo = 1.88) surfaces. The solid squares indicate the IMFPs calculated using the Oswald–Kasper–Gaukler (OKG) theoretical model [21] for the Mo_0.293_S_0.551_C_0.156_ surface. The solid line are values of the fitted function (Equation (1)) to EPES IMFPs. The dashed line shows IMFP values predicted from the TPP-2M formula [19] for the Mo_0.293_S_0.551_C_0.156_ surface.

Figure 4 and Table 1 show comparison of the EPES IMFPs evaluated for the considered surface stoichiometries (in S/Mo AC ratios) and electron energies, and the values resulting from the TPP-2M predictive formula [19] for the Mo_0.293_S_0.551_C_0.156_ (the S/Mo AC ratio of 1.88) and MoS_2_ surfaces. Here are the IMFPs calculated using the OKG theoretical model [21] for the Mo_0.293_S_0.551_C_0.156_ surface. There are also the function fitted to the EPES IMFPs measured for energies between 500 eV and 2000 eV.

The following function was applied in Figure 4 for the fit [10]:

(1) $$\lambda_{fit}=kE^{p}\;$$

where *λ_fit_* is the IMFP obtained from the fit (in angstroms), *E* is the electron energy (in eV) and *k* and *p* are fitting parameters. The resulting values of *k* and *p* are 0.0289 and 0.946, respectively. Fit quality was assessed by calculating the percentage deviation and the mean percentage deviation between the measured and fitted IMFPs, as we explain below.

The IMFP values, *λ*, can be obtained as well from the NIST Database [32] which contains IMFPs calculated from experimental optical data [10,11,12,13,14] and has an option of calculating the IMFPs from the TPP-2M predictive formula [19] for any material, as a function of electron energy *E* in the range from 50 eV to 2000 eV according to:

(2) $$\lambda =\frac{E}{E_{p}^{2}\left[\beta ln\left(\gamma E\right)-\left(C/E\right)+\left(D/E^{2}\right)\right]}$$

(3) $$\beta =-0.10+\frac{0.944}{\sqrt{E_{p\;}^{2}+E_{g}^{2}}}+0.069\rho^{0.1}\;$$

(4) $$\gamma =0.191\rho^{-0.5}\;$$

(5) C=1.97−0.91U 

(6) D=53.4−20.8U 

(7) $$U=\frac{N_{v}\rho }{M}=\frac{E_{p}^{2}}{829.4}$$

where *E_p_* = 28.8(*N_v_ρ*/*M*)^1/2^ is the free-plasmon energy (in eV), *N_v_* is the number of valence electrons per atom (for elemental solids) or molecule (for compounds), *ρ* is the density (in g·cm^−3^), *M* is the atomic or molecular weight and *E_g_* is the band-gap energy (in eV). For stoichiometric MoS_2_, values of *E_g_*, *E_p_*, and *ρ* are 1.8 eV [33,34], 21.72 eV and 5.06 g·cm^−3^ [35], respectively, and *N_v_* = 18 is calculated using the sum of contributions from molybdenum and sulfur. For molybdenum disulfide contaminated by amorphous carbon, *ρ* = 4.75 g·cm^−3^ (Mo_0.315_S_0.581_C_0.104_) or *ρ* = 4.6 g·cm^−3^ (Mo_0.293_S_0.551_C_0.156_) are calculated as a relative contribution of molybdenum disulfide (5.06 g·cm^−3^) and amorphous carbon (*ρ* = 2.1 g·cm^−3^) [35].

As shown in Figure 4 together with the values listed in Table 1, except for the smallest electron energy of 500 eV, experimental values are found to be larger by 0.5–13% than the predicted values, depending on the energy. The differences between IMFPs related to MoS_2_ and carbon-containing Mo disulfide surfaces are estimated to be up to 0.46 nm for electron energies of 500–2000 eV.

We also made comparisons between the experimental and TPP-2M IMFPs and the corresponding IMFPs from the OKG theoretical model [21]. As follows from Figure 4 and Table 1, the latter IMFPs are smaller by 5.1–13.6% and 8.3–22.3% than those obtained from other methods at energies of 1500 and 2000 eV, respectively. In contrast, the IMFP from the OKG calculations has the largest value (1.57 nm) at 500 eV, and it is almost identical to the remaining three IMFPs considered here at 1000 eV.

In order to provide a quantitative description of the fit shown in Figure 4, both the percentage deviation Δ*_j_* from the fitted function and the mean percentage deviation *R_j_* from the fitted function were calculated from:

(8) $$\Delta_{j}=\frac{100\left(\lambda_{j}-\lambda_{fit}\right)}{\lambda_{fit}}\;$$

(9) $$R_{j}=100\frac{1}{r}\overset{r}{\underset{j=1}{\sum }}\left|\frac{\lambda_{j}-\lambda_{fit}}{\lambda_{fit}}\right|\;$$

where *λ_j_* is a measured IMFP for a given Mo disulfide surface at a particular energy, and *r* is the number of IMFP measurements.

Table 2 shows the percentage deviations Δ*_j_* between the measured and fitted IMFPs using Equation (8), as a function of electron energy for each examined surface with respect to the Au standard. The mean percentage deviation *R_j_* from the fitted function, evaluated from Equation (9) (7.11%), is also shown in Table 2. The smallest Δ*_j_* values were found for the surface exhibiting the S/Mo AC ratio of 1.88 at electron energies of 1000 eV (0%) and 1500V (+0.34%), while the largest deviation of +19.09% appear for the disulfide surface with the S/Mo AC ratio of 1.8 at the energy of 1000 eV. The *R_j_* value of 7.11% can be considered acceptably small, so that the fitted function (Equation (1)) was generally consistent with the energy dependence of the EPES IMFPs.

To quantify the comparison of the measured and approximated by Equation (1) IMFPs for the examined Mo disulfide surfaces (solid line in Figure 4) and the IMFPs resulting from the TPP-2M predictive formula [19] for stoichiometric MoS_2_ (Table 1), we calculated the percentage deviation Δ*_f_* and the mean percentage deviation *R_f_*. These deviations, in similarity to Equations (8) and (9), are defined as follows:

(10) $$\Delta_{f}=\frac{100\left(\lambda_{fit}-\lambda_{TPP}\right)}{\lambda_{TPP}}$$

(11) $$R_{f}=100\frac{1}{r}\overset{r}{\underset{j=1}{\sum }}\left|\frac{\lambda_{fit}-\lambda_{TPP}}{\lambda_{TPP}}\right|$$

where *λ_TPP_* denotes the IMFP value estimated from the TPP-2M formula (Equations (2)–(7)) [19] for MoS_2_ at a particular electron energy. Energy dependence of the percentage deviation Δ*_f_* (Equation (10)) and the value of the mean percentage deviation *R_f_* (Equation (11)) are shown in Table 2 as well.

Close inspection of Table 2 reveals the deviation Δ*_f_* to be in the range from +0.5% to −13.44%, and the largest value of this deviation is visible at the electron energy of 500 eV. The *R_f_* value resulting from Equation (11) is equal to 8.77%, and it can be considered acceptably small. Generally, good agreement is found between the measured and approximated by Equation (1) IMFPs and those predicted from the TPP-2M formula [19]. The EPES results prove the high reliability of this formula for the Mo disulfide.

To evaluate the influence of carbon contamination content (up to 15.6 at.%) on the present EPES IMFPs in Mo disulfide, we use values of the information depth (ID) of EPES measurements involving elastic electron backscattering for carbon [18]. These calculations for carbon have been performed at electron energies 0.5–2 keV for the present EPES measurement configuration. Assuming 95% of electron trajectories in a given experimental geometry and for an electron energy of interest, the calculated EPES ID values were found to be 1.11, 1.91, 2.63 and 3.30 nm for energies of 0.5, 1, 1.5 and 2 keV [18]. Except for the lowest energy of 0.5 keV, these values are smaller than IMFPs at energies between 1 keV and 2 keV (Figure 4, Table 1). Therefore, the presence of carbon contamination in the near surface region of Mo disulfide has only a small effect on the EPES-derived IMFP at 0.5 keV (Figure 4, Table 1). We also did not detect any Mo oxides [36].

## 4. Conclusions

In the current work, we characterized thin Mo disulfide crystals obtained using mechanical exfoliation [26,27] and deposited on Si substrates. The surface chemical composition, morphology and electron transport phenomena of prepared Mo disulfide flakes were characterized ex-situ by the combination of AES, SEM and EPES. Prior to the EPES measurements, AES analysis showed that the Mo disulfide overlayer with the thickness exceeding 1.77 nm revealed the maximal AC ratio of S to Mo of 1.88, close to stoichiometric MoS_2_ (2.0). It revealed also the maximal content of about 15 at.% of residual surface carbon. On the basis of SEM results, the presence of single crystalline Mo disulfide flakes exhibiting high crystal quality and thickness uniformity was established. It was found that EPES based on the Au standard can be a useful method for determination of reliable IMFPs in the Mo disulfide.

According to our knowledge, we evaluated for the first time the IMFP values in MoS_2_ with different surface stoichiometry for 0.5–2 keV electrons, using relative EPES measurements. Experimental IMFPs were approximated by a formula: $kE^{p}$, Equation (1), with the parameters *k* = 0.0289 and *p* = 0.946. Experimental IMFPs were compared with the IMFPs resulting from other two sources. Calculations were based on the formalism of the Oswald et al. model [21]. We used also the TPP-2M formula for predicting IMFPs [19]. The EPES IMFPs were found to be in good agreement with the corresponding IMFPs derived from the Oswald et al. model and the TPP-2M predictive formula representing by Equations (2)–(7). Experimental IMFP values were larger by 0.5–13% than the predicted IMFP values in the energy range of 1–2 keV. The *R_f_* deviation from Equation (11) between the EPES IMFPs and the TPP-2M IMFPs was equal to 8.77%. The influence of surface carbon on the EPES IMFPs for molybdenum disulfide was found to be negligibly small (up to 0.46 nm) for electron energies of 0.5–2 keV, and it was the largest at 0.5 keV, as expected.

## Figures and Tables

**Table 1:** The IMFP values (in nm) determined from EPES for Mo disulfide flakes with different surface stoichiometries, the IMFPs estimated from the TPP-2M formula (Equation (2)) [19], and the IMFPs calculated from the OKG theoretical model [21] for the indicated disulfide surface stoichiometries.

IMFP (nm)						
Surface Stoichiometry (in S/Mo AC Ratios)						
	S/Mo = 1.80	S/Mo = 1.84	S/Mo = 1.88		S/Mo = 1.88	S/Mo = 2.00
E (eV)	EPES	EPES	EPES	TPP-2M	OKG theory	TPP-2M
500	0.98	1.07	0.90	1.22	1.57	1.19
1000	2.37	2.31	1.99	2.03	2.03	1.98
1500	2.81	3.16	2.93	2.77	2.57	2.70
2000	3.45	3.75	3.66	3.48	3.13	3.39

**Table 2:** The percentage deviations Δ*_j_* and Δ*_f_*, calculated from Equations (8) and (10) for the considered Mo disulfide stoichiometries (in S/Mo AC ratios) and electron energies. Two final rows show the resulting mean percentage deviations *R_j_* and *R_f_*, calculated from Equations (9) and (11), for the considered surface stoichiometries and electron energies.

Δ*_j_* Deviation (%)				
Energy(eV)	S/Mo = 1.80	S/Mo = 1.84	S/Mo = 1.88	Δ*_f_* Deviation (%)
500	−4.85	3.88	−12.62	−13.44
1000	19.09	16.08	0	0.50
1500	−3.77	8.22	0.34	8.15
2000	−9.92	−2.09	−4.44	12.98

Mean *R_j_* deviation (%): 7.11; Mean *R_f_* deviation (%): 8.77.
